# Nuclear orphan receptor NR2F6 as a safeguard against experimental murine colitis

**DOI:** 10.1136/gutjnl-2016-313466

**Published:** 2017-08-04

**Authors:** Victoria Klepsch, Romana R Gerner, Sebastian Klepsch, William J Olson, Herbert Tilg, Alexander R Moschen, Gottfried Baier, Natascha Hermann-Kleiter

**Affiliations:** 1 Translational Cell Genetics, Department for Pharmacology and Genetics, Medical University of Innsbruck, Innsbruck, Tirol, Austria; 2 Department of Internal Medicine I, Gastroenterology, Endocrinology & Metabolism, Medical University Innsbruck, Innsbruck, Tirol, Austria

**Keywords:** nuclear receptor, colitis, inflammation, intestinal epithelial barrier

## Abstract

**Objective:**

Nuclear receptors are known to regulate both immune and barrier functions in the GI tract. The nuclear orphan receptor NR2F6 has been shown to suppress the expression of proinflammatory cytokines in T lymphocytes. *NR2F6* gene expression is reduced in patients with IBS or UC, but its functional role and tissue dependency in healthy and inflamed gut have not yet been investigated.

**Design:**

Intestinal inflammation was induced in wild-type, *Nr2f6*-deficient, *Rag1*-deficient or bone marrow-reconstituted mice by administration of chemical (dextran sodium sulfate (DSS)) and immunogenic (T cell transfer) triggers. Disease phenotypes were investigated by survival, body weight, colon length and analysis of immune cell infiltrates. Additionally, histology, intestinal permeability, tight junction proteins, bacterial fluorescence in situ hybridisation, apoptosis, cell proliferation and mucus production were investigated.

**Results:**

*Nr2f6*-deficient mice were highly susceptible to DSS-induced colitis characterised by enhanced weight loss, increased colonic tissue destruction and immune cell infiltration together with enhanced intestinal permeability and reduced *Muc2* expression. T cell transfer colitis and bone marrow reconstitution experiments demonstrated that disease susceptibility was not dependent on the expression of *Nr2f6* in the immune compartment but on the protective role of NR2F6 in the intestinal epithelium. Mechanistically, we show that NR2F6 binds to a consensus sequence at −2 kb of the *Muc2* promoter and transactivates *Muc2* expression. Loss of NR2F6 alters intestinal permeability and results in spontaneous late-onset colitis in *Nr2f6*-deficient mice.

**Conclusion:**

We have for the first time identified a fundamental and non-redundant role of NR2F6 in protecting gut barrier homeostasis.

**Significance of this study**

**What is already known on this subject?**

- Nuclear receptor subfamily 2, group F, member 6 (NR2F6) potently antagonises the ability of T helper 17 CD4^+^ T cells to induce the expression of *Il17* and thus suppresses autoimmunity.
- NR2F6 is an intracellular immune checkpoint, directly repressing transcription of cytokine genes in T cells relevant for cancer cell rejection, such as interleukin-2, interferon γ and tumour necrosis factor α.
- NR2F6 is highly expressed by intestinal epithelial cells, and low *NR2F6* expression status has been observed in patients with IBD and UC.

**What are the new findings?**

- *Nr2f6^−/*−*^* mice show increased susceptibility to DSS-induced colitis compared with wild-type mice, characterised by an aggravated clinical disease phenotype and enhanced immune cell infiltration.
- *Nr2f6^−/−^* CD4^+^ T cells are not the primary cause of increased colonic inflammation and disease pathology. Rather, loss of NR2F6 in colon epithelial cells enhanced intestinal permeability, leading to spontaneous colitis in 1-year-old *Nr2f6*-deficient mice.
- Mechanistically, NR2F6 directly transactivates *Muc2* expression via binding to its consensus site at −2 kb of the *Muc2* promoter in human colon carcinoma cell line LoVo and primary mouse colon epithelial cells.

**How might it impact on clinical practice in the foreseeable future?**

- These findings support the idea that selective agonists of NR2F6 might represent a novel therapeutic strategy in the treatment of certain forms of human IBD, especially as nuclear receptors are well-known drug targets.

## Introduction

Crohn’s disease and UC, the two main forms of IBD, affect over 2.5 million people of European ancestry, with rising prevalence in other populations.^1^ IBD is thought to occur as a result of the complex interplay among host genetics, environmental factors such as gut microbiota and nutrition, and the host immune system.^2^ Proinflammatory cytokines are known to play a central role in the pathogenesis of intestinal inflammation, resulting in an enhanced inflammatory potential of immune cells and further decreasing barrier function and self-renewal properties of the intestinal epithelium, thus exacerbating inflammation.^3–5^

Within the GI tract, nuclear receptors (NRs) are well-known sensors of hormones, namely, oestrogen receptor (ER) or glucocorticoid receptor (GCR), nutrients such as vitamin A and retinoic acid receptor (RAR), vitamin D and vitamin D receptor (VDR) and certain host-bacterial metabolites such as bile acid and farnesoid X receptor (FXR), indoles and pregnane-X-receptor (PXR),^6^ linoleic acid and peroxisome proliferator-activated receptor γ (PPARγ).^7 8^ Several NRs as PPARγ, VDR, RARα, GCR, FXR, ER-β or hepatocyte nuclear factor 4 (Hnf4) α have been shown to play fundamental roles in epithelial intestinal cell integrity, modulating different mechanisms ranging from sensing microbial metabolites, regulating mucus secretion, goblet cell loss and autophagy or regulating tight junction protein expression and localisation.^9–16^

NRs also contribute to gut homeostasis by shaping intestinal immune cells that are constantly challenged in the face of stimulation by gut microbiota. Especially the reciprocal differentiation potential of naive CD4^+^ T cells into either proinflammatory T helper 17 (Th17) or regulatory T cells is shaped by several NRs such as PPARγ, RAR, VDR, liver X receptor (LXR), NR subfamily four group A member 2 or RAR-related orphan receptor γ (RORγ) (see recent review).^15^ In addition, innate lymphoid cells expressing the nuclear receptor RORγ or RORα as well as macrophages expressing PPARγ, NR4A1 or LXR are essential for gut immune homeostasis.^17 18^

NR2F family members homodimerise or heterodimerise with retinoid X receptor (RXR/NR2B1) as well as other NRs and bind to a variety of response elements that contain imperfect AGGTCA direct or inverted repeats with various spacing on the cognate DNA sequence; a natural ligand has not yet been identified.^19^ Members of the nuclear orphan receptor chicken ovalbumin upstream promoter-transcription factor (COUP) family NR2F1 (COUPTF-I), NR2F2 (COUPTF-II) and NR2F6 (COUPTF-III; Ear2) are highly abundant in the healthy proximal colon of both mice and humans, but the functional role of NR2F6 has not been investigated.^20^ In contrast to a high expression status in healthy intestinal epithelial cells, downregulation of *NR2F6* expression has been reported by several studies on human patients with colitis or IBD within the relevant expression data sets.^21–25^ We have previously shown that the orphan NR subfamily 2, group F, member 6 (NR2F6) represents an important gatekeeper of antigen receptor-induced response thresholds of proinflammatory cytokines as interferon γ (IFNγ), tumour necrosis factor α (TNFα) and interleukin 17 (IL-17) in T cells.^26–28^

On the one hand, NR2F6 is an adaptive immune regulator keeping proinflammatory cytokine responses in check, but on the other hand, it is also highly expressed by intestinal epithelial cells. Therefore, we believed that the potential role of NR2F6 in the regulation of GI homeostasis was worth investigating.

For this purpose, we examined colitis disease phenotypes of dextran sodium sulfate (DSS)-treated, T cell transfer-induced and bone marrow (BM)-reconstituted animals. We found *Nr2f6^−^*^/−^ and wild-type BM-reconstituted *Nr2f6*^−^*^/−^* mice to be highly susceptible to DSS-induced colitis, whereas *Nr2f6*^−^*^/−^* BM-reconstituted wild-type and wild-type mice showed a less severe phenotype. In agreement with these observations, the severity of colitis in T cell-dependent transfer experiments was not different between genotypes. Thus, loss of NR2F6 in the intestinal epithelium appears to be the primary cause of enhanced disease susceptibility in *Nr2f6*-deficient mice, validating for the first time NR2F6 as protective player in the counter-regulation of intestinal inflammation.

## Methods

### Mice

Female *Nr2f6*-deficient mice of 6–12 weeks old 29 back-crossed on a C57BL/6 background or wild-type animals were used for experiments. *Rag1*^−^*^/−^* (B6.129S7-*Rag*1^tm1Mom^/J) mice were provided by ARM and used for T cell transfer colitis induction.

### DSS colitis induction

Colitis was induced in wild-type and *Nr2f6*-deficient littermates with 3.5% DSS (molecular weight 36 000–50 000; MP Biomedicals) dissolved in drinking water given ad libitum for 5 consecutive days followed by a 2-day tap water period.^30 31^ BM chimeric mice (*Nr2f6^+/+^* mice with *Nr2f6^+/+^* or *Nr2f6^–/–^* BM or *Nr2f6^–/–^* mice with *Nr2f6^+/+^* or *Nr2f6^–/–^* BM) were treated with 2.5% DSS for 5 days followed by 2 days of tap water after an 8-week postirradiation recovery phase.

### In vivo barrier function experiments

Wild-type or *Nr2f6^–/–^* mice of 8–12 weeks old were gavaged with 0.6 mg/g body weight of an 80 mg/mL solution of fluorescein isothiocyanate (FITC) dextran (Sigma Aldrich, St. Louis, Missouri, USA), and serum was collected after 4 hours. A standard curve was prepared using serial dilutions of dextran in phosphate-buffered saline. Fluorescence emission was measured on a PHERAstar plus microplate reader (BMG Labtech, Ortenberg, Germany) at an excitation of 485 nm and an emission of 521 nm.

### Chromatin immunoprecipitation

Chromatin immunoprecipitation (ChIP) assay was performed with a ChIP assay kit according to the recommendations of the manufacturer (Chromatrap ChIP SEQ kits Premium/Chromatrap pro G ChIP spin column kit 24 (500190)) and previously described methods.^28^ Human colon carcinoma cell lines LoVo or Caco-2 were grown in Dulbecco’s modified Eagle medium GlutaMax 4.5 g/L glucose (Gibco, 31966) medium+10% fetal calf serum, penicillin/streptomycin, HEPES and sodium bicarbonate. Colons of healthy or DSS-diseased mice were individually scraped into X-vivo medium and fixed for 6 min at room temperature in 1% formaldehyde followed by 5 min glycine (0.65 M) quenching. Cells were lysed, and subsequently, sonication was performed with 18×30 s pulses using a Bioruptor Next Generation (Diagenode). The sheared chromatin was used to set up immunoprecipitation reactions with 5 µg of the indicated antibodies (IgG2b, BioXell; NR2F6, Perseus Proteomics, Histone H3Ac, H3K27me3, Millipore)^27 28^ at 4°C for 1 hour in a 2:1 antibody:chromatin ratio. Immunoprecipitation was subsequently performed with Chromatrap spin columns followed by reverse cross-linking and a clean-up of the DNA with Qiagen MinElute columns. Real-time PCR was performed using an ABI PRIM 7000 Sequence Detection System (Applied Biosystems) with the following primers and probes: mouse Muc2: ChIP_Sense −2057 5′-GGTCATTCAGCTTGGGTCAC-3′; ChIP_Antisense 5′-CGGATGGAGGGAGTAGATCC-3′ −1948 (product length 114). Human primers were used according to Yamada *et al*.^32^

### RNA isolation and gene expression analysis

Colon scrapings were collected on ice and stored for a short term at −80°C. Total RNA was isolated using the RNeasy Plus Mini Kit (Qiagen). First-strand cDNA synthesis was performed using oligo(dT) primers (Promega) with the Qiagen Omniscript RT kit, according to the instructions of the supplier and as described previously.^27^ Expression analysis was performed using real-time PCR with an ABI PRIM 7000 or ABI PRIM 7500 Fast Sequence Detection System with TaqMan gene expression assays (Applied Biosystems); all expression patterns were normalised to GAPDH.

### Histological analysis

Detailed methods regarding DSS colitis analysis, histological procedures and scoring, Swiss roll histology, immunohistochemistry, isolation of lamina propria (LP) lymphocytes, flow cytometry, BM chimeras, transfer colitis model, in vivo 5-bromo-2-deoxyuridine (BrdU) labelling, analysis of apoptosis and bacterial fluorescence in situ hybridisation (FISH) can be found in the  online supplementary Material and methods section.

10.1136/gutjnl-2016-313466.supp1 Supplementary material 1

### Statistical analysis

Data were analysed using Prism 5.03 software (GraphPad Software). Experiments were repeated at least two times. Data are represented as indicated (either the mean±SEM or ±SD) for all figure panels in which error bars are shown. The p values were assessed using two-tailed unpaired Student’s t-test, log-rank test or analysis of variance. A p value of less than 0.05 was considered statistically significant (*p<0.05; **p<0.01; ***p<0.001).

## Results

### *Nr2f6*^–/–^** mice are highly susceptible to DSS-induced colitis

**Figure 1:**
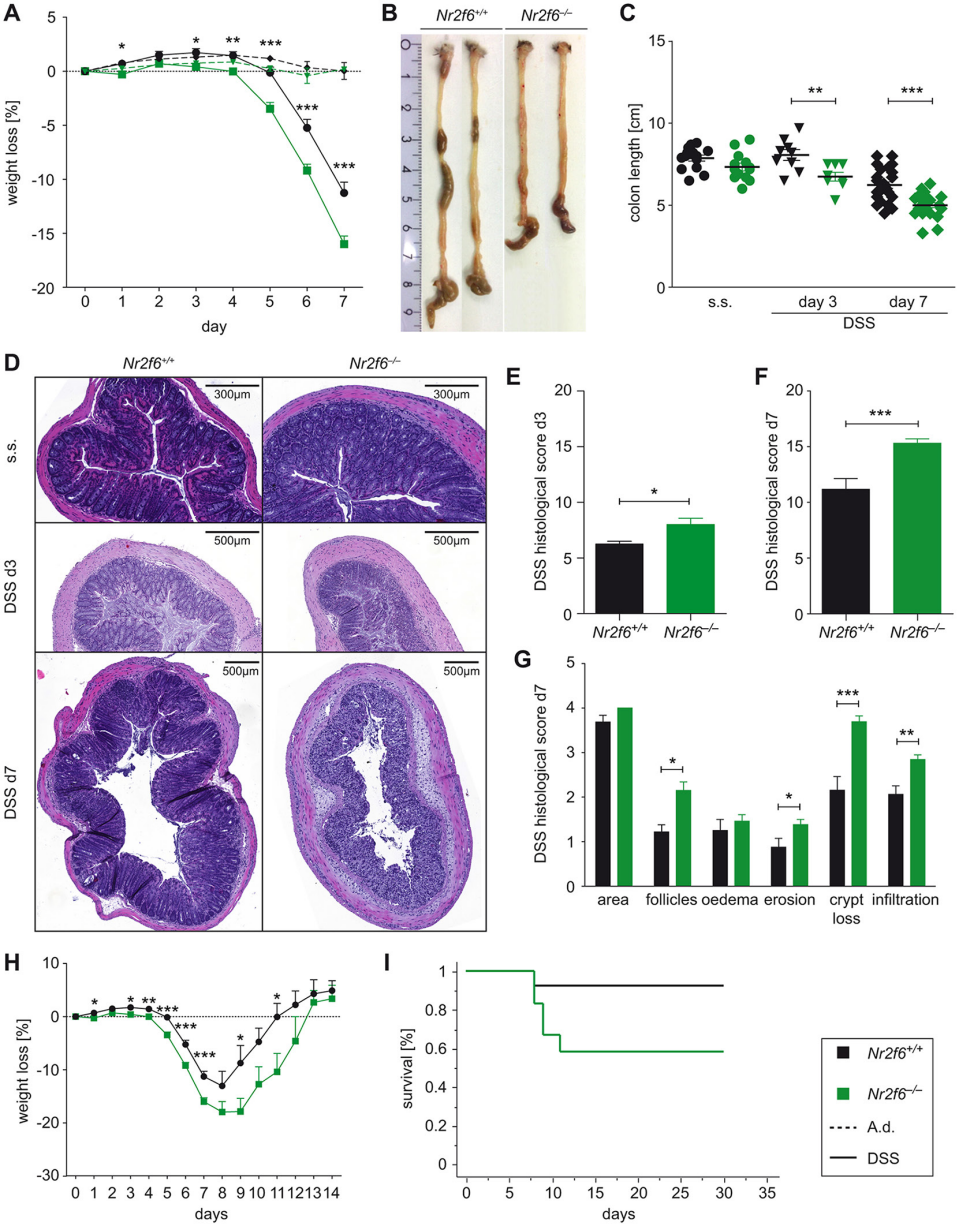
*Nr2f6*^−/−^ mice are highly susceptible to dextran sodium sulfate (DSS)-induced colitis. (A) Weight loss in *Nr2f6*^+/+^ or *Nr2f6*^−/−^ mice relative to initial weight treated with 3.5% DSS ad libitum, followed by 2 days of tap water (n=35, analysis of variance p<0.0001; t-test d3 p=0.01, d4 p=0.004, d5 p<0.0001, d6 p=0.0002, d7 p=0.0003). (B) Representative pictures of colons on day 7 after DSS induction. (C) Colon length of wild-type and *Nr2f6^−/−^* mice on day 0 (steady state (s.s.) day 3 (n=9, p=0.0081) and day 7 (n=25, p<0.0001) after DSS treatment. (D) H&E staining of colon sections of wild-type and *Nr2f6*-deficient mice at different time points (s.s., days 3 and 7). Histological severity scores of respective groups are shown on day 3 (p=0.027) (E) and day 7 (p=0.0005) (F) after DSS treatment. (G) Note the disruption of crypt structure (p=0.0002), erosion (p=0.048), follicle aggregation (p=0.009) and immune cell infiltration (p=0.0024) in *Nr2f6^−/−^* mice on day 7. (H) Weight loss curve of wild-type and *Nr2f6^−/−^* mice treated with 3.5% DSS for 5 days following a recovery phase with water from days 5 to 14. (I) Kaplan-Meier survival analysis of recovery mice showing 92.3% (12 out of 13) wild-type survivors compared to 58.3% (7 out of 12) surviving *Nr2f6*-deficient mice (p=0.046). Data are presented as mean±SEM error bars and are representative of at least two independent experiments if not stated otherwise. Unpaired Student’s t-test, * p<0.05.

In order to investigate the role of NR2F6 in experimental colitis disease progression, we administered 3.5% DSS in the drinking water to wild-type as well as *Nr2f6*-deficient mice. Loss of NR2F6 led to rapid onset and accelerated progression of disease as assessed by overall weight loss and colon length (figure 1A–C) on days 3 and 7 after DSS exposure. Histological examination and scoring of distal colon sections on days 3 and 7 revealed a significantly higher pathology score characterised by increased epithelial disruption, follicle aggregation, enhanced erosion, increased crypt loss and increased infiltration of immune cells in *Nr2f6*-deficient animals (figure 1D–G). Analysis of Swiss roll colon sections on day 7 revealed that especially the colonic epithelial disruption of *Nr2f6^−/*−*^* animals reached far more proximal compared with wild-type mice after DSS administration (see online supplementary figure S1A). Survival during the recovery phase (days 8–14) of *Nr2f6*-deficient mice was impaired as 41.7% of *Nr2f6^−/−^* mice (7 out of 12) had to be sacrificed between days 8 and 12 during the experiment due to >20% weight loss in comparison to 7.7% of wild-type (1 out of 13) mice (figure 1H,I). In addition, recovery was delayed 1 day as *Nr2f6*-deficient mice started to gain weight on day 10 (−17.8% mean weight reduction of *Nr2f6^−/−^* mice while wild-type lost −8.7% of initial body weight on day 9); wild-type mice already gained weight on day 9 (figure 1H). Within the surviving cohort, relative weight gain between wild-type (days 8–11) and *Nr2f6*-deficient mice (days 9–12) was comparable between genotypes (figure 1H).

In order to detect differences in the apoptosis rate of *Nr2f6^−/−^* and wild-type colonic epithelial cells, steady-state epithelial tissues were analysed by terminal deoxynucleotidyl transferase dUTP nick end labelling; however, no significant differences were observed in epithelial cell apoptosis rates between genotypes (see online supplementary figure S1B, C).

To examine whether loss of NR2F6 might regulate proliferation in steady state or in the inflamed colon (d3), we injected naive and DSS-treated mice with BrdU and sacrificed the animals 2 and 24 hours later. Staining with BrdU-specific antibody as well as expression analysis of the proliferation marker Ki-67 did not reveal any significant differences in basal crypt proliferation rates between naive wild-type and *Nr2f6^−/−^* mice (see online supplementary figure S1D–F).

Epithelial self-renewal properties and differentiation of the colonic epithelium in *Nr2f6*-deficient colons were investigated by expression analysis of stem (*Lgr5*, *Ascl2*), enteroendocrine (*Chga*), goblet (*Tff3*, *Clca1*), enterocyte (*Alpi*) and tuft (*Dclk1*) cells in steady state; no significant differences were detected between genotypes (see online supplementary figure S1G).

### Loss of NR2F6 enhances infiltration of immune cells during DSS colitis

**Figure 2:**
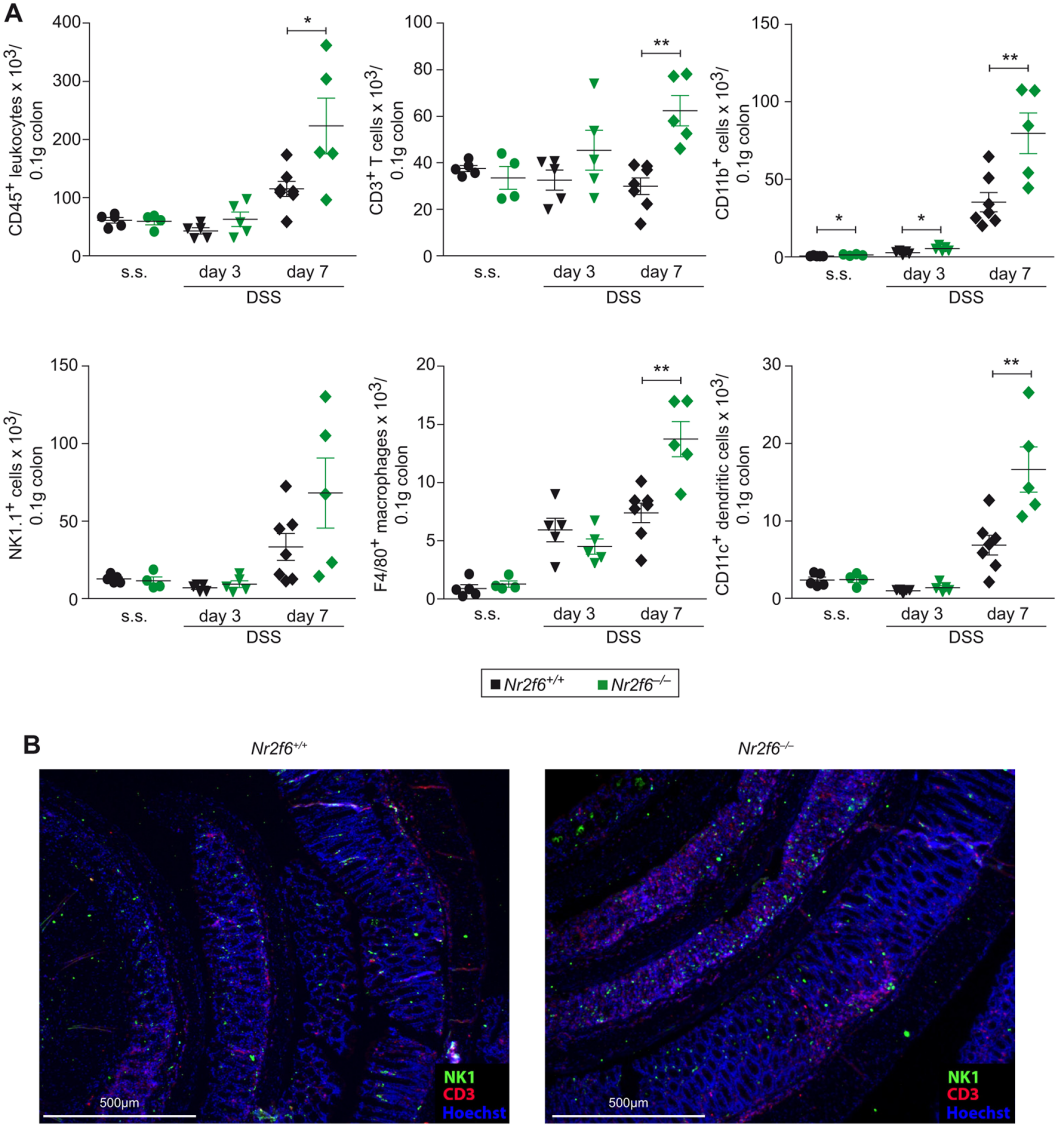
Enhanced immune cell infiltration into the colon of *Nr2f6^−/−^* diseased mice. (A) Colon-infiltrating cell numbers per 0.1 g of tissue of wild-type and *Nr2f6^−/−^* mice were stained for CD45^+^ leucocytes (p=0.029, day 7), CD3^+^ T cells (p=0.0008, day 7), CD11b^+^ cells (p=0.023, day 0; p=0.022, day 3; p=0.0072, day 7), NK1.1^+^ natural killer cells, F4/80^+^ macrophages (p=0.0028, day 7) and CD11c^+^ dendritic cells (p=0.0065, day 7) at indicated time points (n=5–7). (B) Representative immunofluorescence staining for CD3^+^ T cells (red), NK1.1^+^ natural killer cells (green) and Hoechst nuclear stain (blue) of wild-type and *Nr2f6^−/−^* Swiss rolls (n=5). Data are presented as mean±SEM error bars and are representative of at least two independent experiments. Unpaired Student’s t-test, * p<0.05.

To determine the effects of *Nr2f6* ablation on intestinal immune cellularity, we isolated cells from the LP of steady state, day 3 and day 7 DSS-diseased wild-type and *Nr2f6^−/−^* mice and analysed them by flow cytometry. Significantly increased numbers of CD45^+^ leucocytes, CD3^+^ T cells, CD11b^+^ cells as well as NK1.1^+^ natural killer cells, F4/80^+^ macrophages and CD11c^+^ dendritic cells infiltrated the colonic LP of *Nr2f6*-deficient mice on day 7 when compared with wild-type controls (figure 2A) (see online supplementary figure S2). These findings were corroborated by immunohistochemistry on colonic Swiss roll sections showing enhanced infiltration of CD3^+^ T cells in *Nr2f6*-deficient colonic epithelium on day 7 after DSS induction (figure 2B).

### Naive *Nr2f6*-deficient CD4^+^ T cells do not exaggerate transfer colitis

We have previously shown that NR2F6 impacts proinflammatory cytokine expression in activated T cells. In order to determine whether enhanced inflammation and tissue destruction of the epithelial barrier during DSS colitis is causative of enhanced activation-dependent potential of *Nr2f6^−/−^* CD4^+^ (CD25^−^CD62L^hi^CD44^lo^) T cells, we used a model of T cell-dependent colitis. The transfer colitis model into recombination-activating gene 1 (*Rag1^−/−^)*-deficient recipients which lack T and B cells was used.^2^

**Figure 3:**
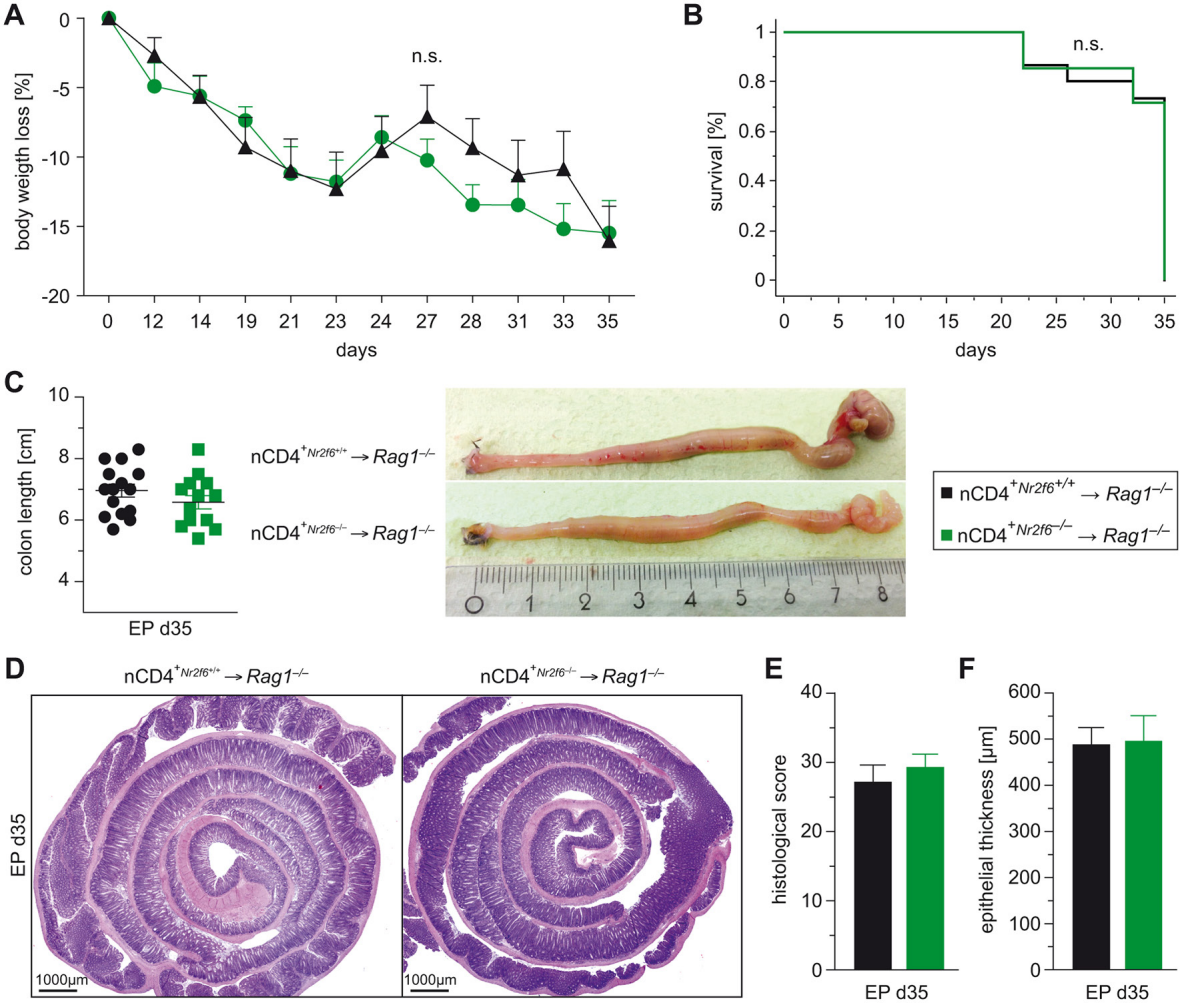
*Nr2f6^−/−^* CD4^+^ T cells do not induce enhanced colitis in *Rag1^−/−^* mice. (A) Body weight, (B) Kaplan-Meier survival curve and (C) colon length of *Rag1^−/−^* recipient mice receiving adoptive cell transfer of 5×105 naive (CD25^-^CD62L^hi^CD44^lo^) CD4^+^ T cells isolated from peripheral lymphatic organs of wild-type or *Nr2f6^−/−^* mice (n=15). (D) Representative H&E-stained colonic Swiss rolls, (E) histological severity scores and (F) epithelial thickness of *Rag1^−/−^* recipients adoptively transferred with either wild-type or *Nr2f6^−/−^* naive CD4^+^ T cells (nCD4^+^). Data are presented as mean±SEM error bars and are representative of at least two independent experiments. Unpaired Student’s t-test, * p<0.05.

Surprisingly, disease severity documented by survival, weight course and histological analysis of colons was comparable between wild-type and *Nr2f6^−/−^* naive CD4^+^-reconstituted *Rag1*-deficient recipient mice, suggesting no difference in the inflammatory potential of CD4^+^ effector cells between genotypes within the *Rag1*-deficient intestinal microenvironment over time (figure 3A–F).

### Immune cell-derived NR2F6 does not protect against DSS colitis in BM-reconstituted mice

**Figure 4:**
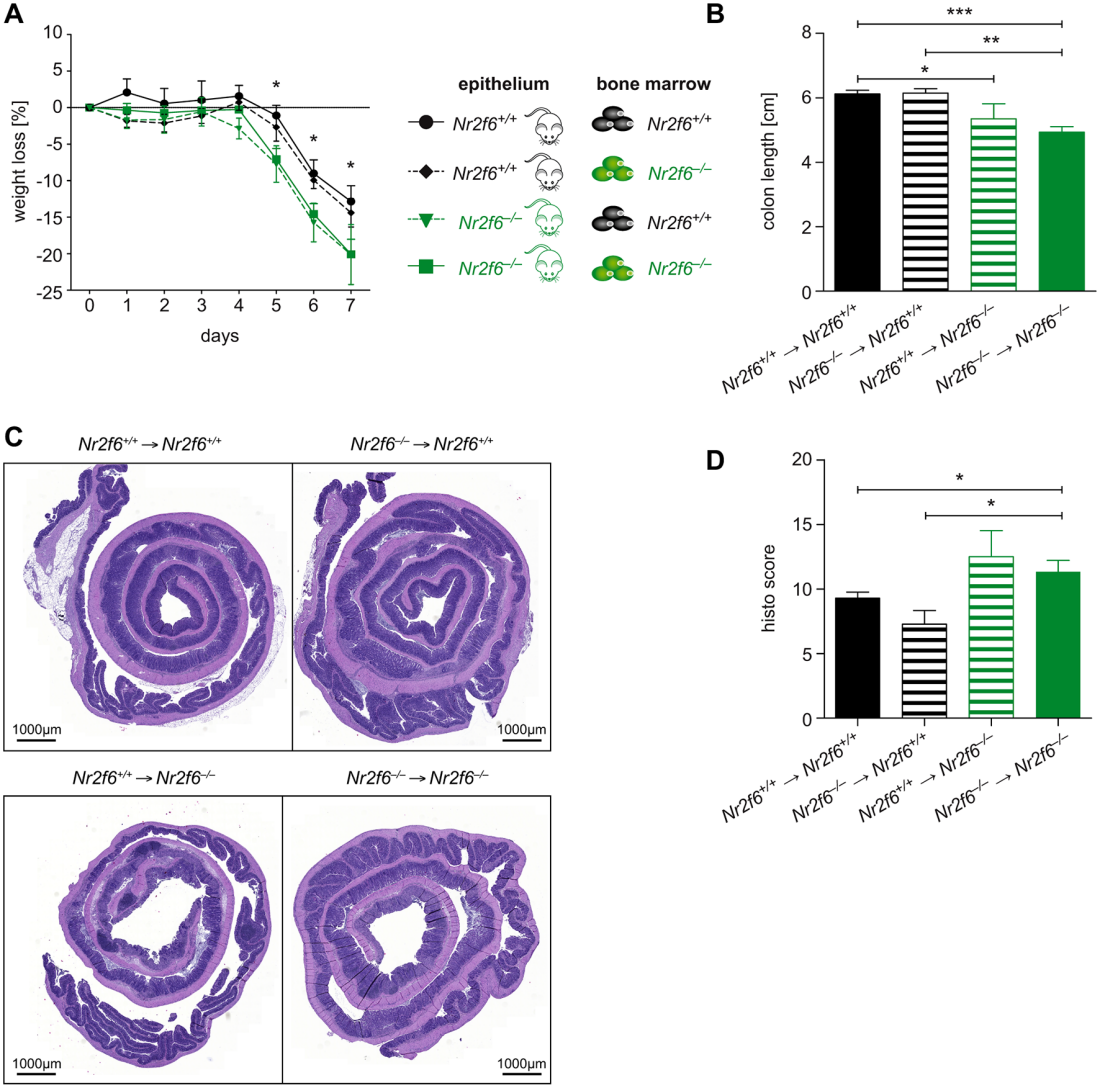
NR2F6 in intestinal epithelial cells, not immune cells, protects against dextran sodium sulfate (DSS) colitis. (A) Bone marrow (BM) chimeras were generated using *Nr2f6*^+/+^ and *Nr2f6*^−/−^ mice as recipient and BM donor. Body weight loss is more severe in mice with *Nr2f6*^−/−^ epithelium regardless of immune system origin (n=6–10) (exemplary p values are given for day 5 wild type (wt) versus *Nr2f6*^−/−^ d5 p=0.013; wt versus wt BM in *Nr2f6*^−/−^ mice d5 p=0.029; *Nr2f6*^−/−^ BM in wt mice versus *Nr2f6*^−/−^ d5, p=0.027). (B) Mean colon lengths from indicated genotypes (wt versus wt BM in *Nr2f6*^−/−^ mice p=0.044; wt versus *Nr2f6*^−/−^ p<0.0001; *Nr2f6*^−/−^ BM in wt mice versus *Nr2f6*^−/−^ p=0.001). (C) Representative H&E-stained sections of colonic Swiss rolls of *Nr2f6*^+/+^ and *Nr2f6*^−/−^ reconstituted mice. (D) Histological colitis severity was scored 8 weeks after BM transplantation and treatment with 2.5% DSS for 5 days, on day 7 after induction (wt versus *Nr2f6*^−/−^ p=0.039; *Nr2f6*^−/−^ BM in wt mice versus *Nr2f6*^−/−^ p=0.024) according to parameters described in the supplementary Material and methods section and also as previously specified.^33^ ^44^ Data are presented as mean±SEM error bars and are representative of at least two independent experiments. Unpaired Student ’s t-test, * p<0.05.

To directly answer the question whether the initial signals leading to enhanced inflammation and tissue destruction of the epithelial barrier during DSS colitis originate in haematopoietic or non-haematopoietic cells (eg, predominantly the epithelium), we performed BM chimera experiments with *Nr2f6*^+/+^ and *Nr2f6*^−/−^ mice. These studies clearly showed that immune reconstitution with either *Nr2f6*^+/+^ or *Nr2f6*^−/−^ haematopoietic cells had no effect on DSS disease progression in *Nr2f6*^+/+^ mice. In contrast, immune reconstitution with either *Nr2f6*^+/+^ or *Nr2f6*^−/−^ haematopoietic cells into *Nr2f6*^−/−^ mice resulted in enhanced weight loss, diarrhoea, reduced colon length and aggravated disease scores (figure 4A–D). Together, these data strongly support a crucial role of NR2F6 in non-haematopoietic cells, conceivably intestinal epithelial cells.

### Gut epithelial integrity is compromised in the absence of NR2F6 due to reduced *Muc2* expression resulting in defective barrier function

**Figure 5:**
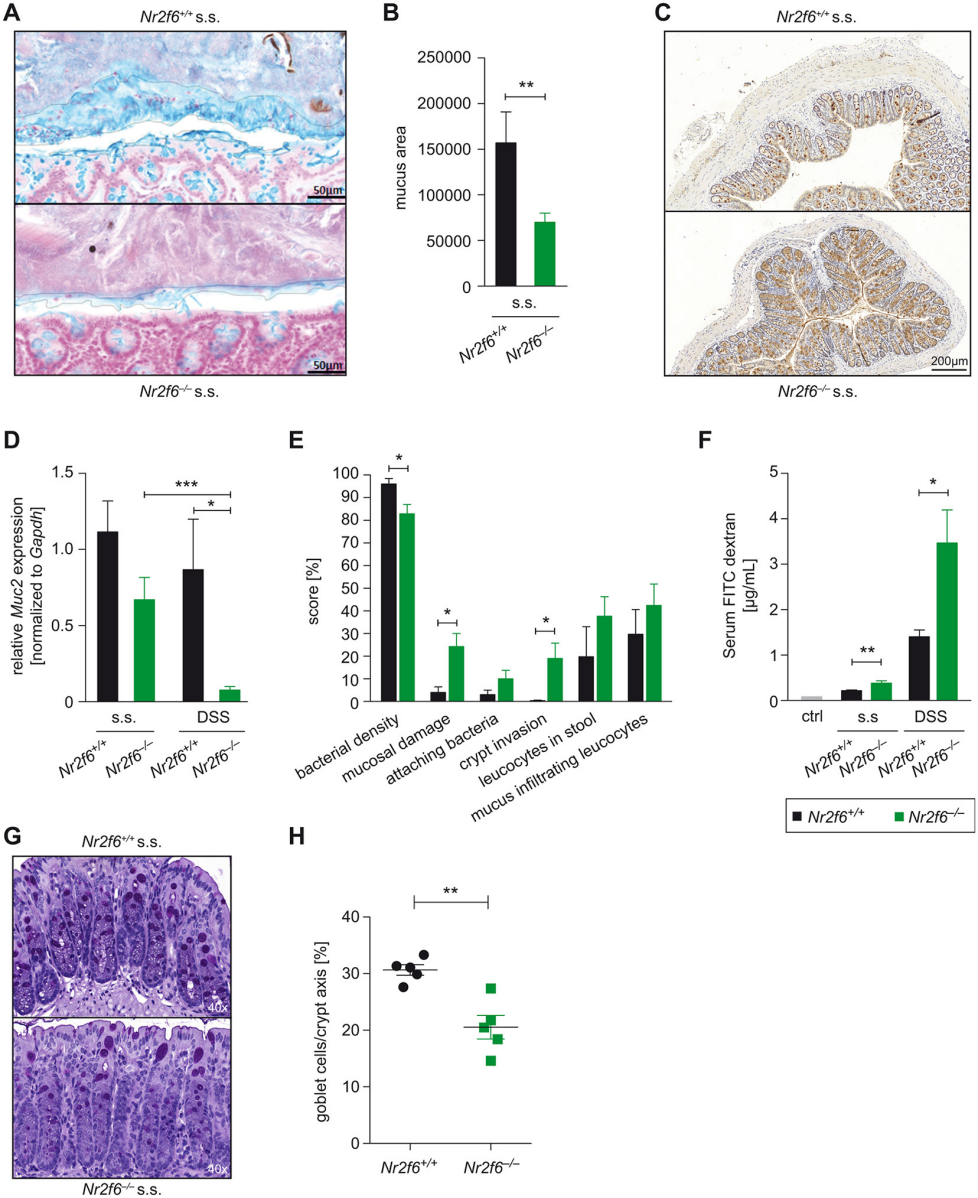
*Nr2f6*^−/−^ colons have impaired mucus barrier integrity. (A) Steady-state (s.s.) 10- to 12-week-old wild-type and *Nr2f6*^−/−^ colonic tissues were fixed with Carnoy’s fixative, sectioned, stained with Alcian blue (blue) and (B) analysed calculating the mucus area using ImageJ (n=8, p=0.0091). (C) Representative images of immunohistochemical staining (brown) with Muc2 antibody in colon sections of steady-state *Nr2f6*^+/+^ and *Nr2f6*^−/−^ mice (n=8). (D) Relative amount of Muc2 mRNA detected in colonic scrapings by quantitative real-time PCR (*Nr2f6^−/−^* s.s. versus DSS p=0.0002; wild type versus *Nr2f6*^−/−^ DSS p=0.021). Data are expressed as relative fold change between *Nr2f6*^+/+^ and *Nr2f6*^−/−^ mice at baseline (n=8–11). (E) Colon sections of 10-week-old s.s. mice were probed with an Alexa Fluor 555-conjugated pan-bacterial EUB338 probe for fluorescence in situ hybridisation (FISH) (n=8) and scored for the indicated features as described in detail in the online supplementary Material and methods section. Data are presented as percent involvement according to the respective genotype (n=8; bacterial density p=0.036, mucosal damage p=0.017, crypt invasion p=0.04). (F) Serum concentrations of fluorescein isothiocyanate (FITC) dextran were measured 4 hours after oral administration to *Nr2f6*^+/+^ and *Nr2f6*^−/−^ mice in the s.s. as well as on day 7 after DSS induction (n=5 10; s.s. p=0.005, DSS p=0.022). (G) Representative images of PAS-stained histological colon samples from healthy 10-week-old *Nr2f6*^+/+^ and *Nr2f6*^−/−^ mice showing significantly decreased goblet cell counts per crypt (p=0.0023) in *Nr2f6-*deficient mice (H). Data are presented as mean±SEM error bars and are representative of at least two independent experiments. Unpaired Student’s t-test, * p<0.05.

Neither the expression nor the spatial organisation of tight and adherens junction proteins such as E-cadherin, occludin or ZO-1 was changed in steady-state *Nr2f6*-deficient colonic epithelium when compared with wild type (see online supplementary figure S3A–D). Subsequently, we investigated the colonic mucus layer on Carnoy-fixed, Alcian blue-stained colon tissue sections. Quantification of the mucus-covered interlaced area bordered by apical epithelial cells on one side and faeces on the other side revealed a significant decrease of the mucus-covered area already present in colon sections of young steady-state *Nr2f6*^−/−^ mice when compared with wild type (figure 5A, B).

The major component of the colonic mucus layer is the gel-forming mucin 2 (Muc2); accordingly, we investigated whether the transcription factor NR2F6 impacts *Muc2* expression. Indeed, the intensity of anti-Muc2-stained mucus layer was reduced in *Nr2f6^−/−^* colons in the steady state when compared with wild type (figure 5C). On a transcriptional level, *Muc2* mRNA expression was reduced in steady-state *Nr2f6^−/−^* colon specimens, although not significantly, but a strongly reduced *Muc2* mRNA expression level in *Nr2f6^−/−^* colons was observed on day 7 on DSS treatment-induced intestinal inflammation (figure 5D).

16S rRNA FISH with the pan-bacterial EUB338 probe revealed that wild-type colon epithelium was covered by an intact mucus layer that was devoid of bacteria, whereas *Nr2f6^−/−^* epithelium showed an impaired mucus layer with bacteria in direct contact with the epithelial surface when scored as previously described (figure 5E).^33^ Expression analysis of additional members of the *Muc* gene family, namely, *Muc1, 3, 4, 5ac* and *6*, was investigated in healthy wild-type and *Nr2f6^−/−^* colonic scrapings; no significant differences for *Muc3*, *4* and *5ac* were observed between genotypes, and *Muc6* expression was not detectable in either genotype. *Muc1* expression was significantly reduced in colonic scrapings from healthy *Nr2f6*-deficient mice. During DSS-induced inflammation, significantly reduced *Muc3* expression could be detected in *Nr2f6*-deficient colonic scrapings after DSS induction (see online supplementary figure 3E). The breached mucus integrity was associated with higher intestinal barrier permeability, as FITC-dextran-fed *Nr2f6^−/−^* mice demonstrated increased serum concentrations of FITC dextran already at steady state as well as on day 7 after DSS administration (figure 5F). Additionally, loss of NR2F6 resulted in significant although partial goblet cell loss in steady-state colons (figure 5G,H) when compared with wild-type mice.

**Figure 6:**
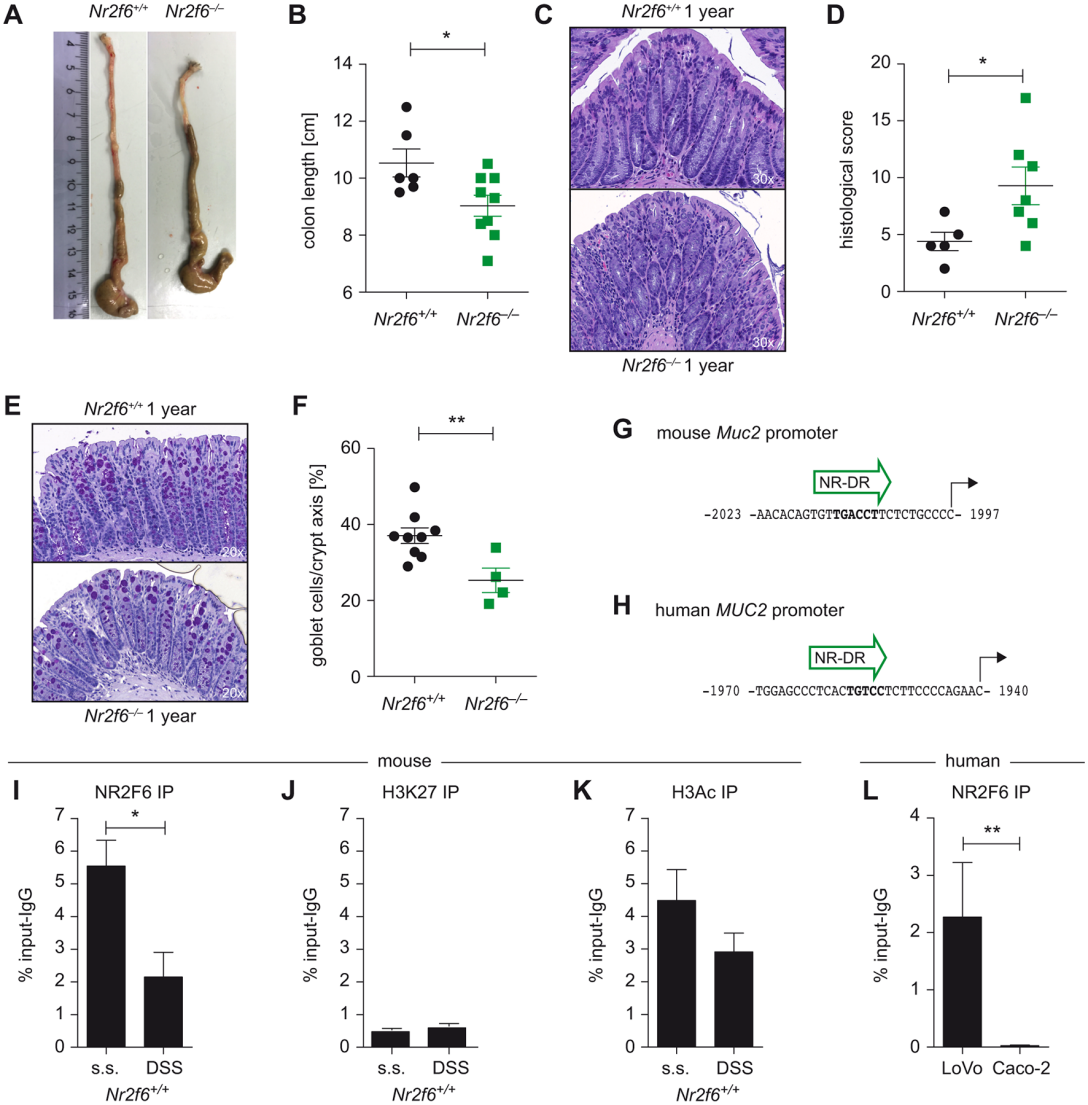
*Nr2f6^−/−^* mice develop spontaneous colitis, and NR2F6 directly binds to the Muc2 promoter. (A) Representative pictures of colons of wild-type and *Nr2f6^−/−^* 1-year-old female mice with significant shortened colon lengths in *Nr2f6^−/−^* mice (n=9) when compared to wild-type (n=6) controls (p=0.027). (C) H&E staining (D) and histological severity scores of colon sections of 1-year-old wild-type and *Nr2f6*-deficient mice (p=0.042). (E) Representative images of PAS-stained histological colon samples from 1-year-old *Nr2f6*^+/+^ and *Nr2f6^−/−^* mice showing significantly decreased goblet cell counts per crypt (p=0.007) in *Nr2f6*-deficient mice (F). (G) A putative nuclear receptor direct repeat site (NR-DR) site at −2 kb of the mouse and (H) human MUC2 gene as predicted by TRANSFAC software is shown. (I) Chromatin immunoprecipitation (ChIP) PCR of the mouse −2 kb promoter region was performed from healthy or DSS diseased (day 3) wild-type colon scrapings with subsequent immunoprecipitation with NR2F6, (K) H3Ac, (J) or H3K27 antibodies. NR2F6 binding was significantly lower (p=0.0362) in the diseased mice, whereas H3Ac or H3K27 binding capability did not change between the two groups (n=3) but was significantly different between the marker for open (H3Ac) and repressed (H3K27) chromatin (p=0.0143). (L) ChIP PCR of the −2 kb huMUC2 promoter region was performed in the human colon carcinoma cell lines LoVo and Caco-2; chromatin was immunoprecipitated with anti-NR2F6; functional binding was significantly lower (p=0.0062) in the low MUC2-secreting Caco-2 colon carcinoma cell line when compared to the high MUC2 LoVo cell line. Data are shown as specific NR2F6 enrichment minus unspecific IgG control (n=4). Data are presented as mean±SEM error bars and are representative of at least two independent experiments. Unpaired Student’s t-test, * p<0.05.

A hallmark of penetrable inner mucus layer is the development of spontaneous colitis.^34^ Therefore, we analysed 1-year-old mice for signs of spontaneous colitis and could indeed document shortened colons, elevated histological score of colitis as well as reduced goblet cell numbers in *Nr2f6^−/−^* mice when compared with age-matched controls (figure 6A–F).

In general, NRs have been shown to regulate mucus gene expression. Using TRANSFAC Transcription Factor Binding Sites prediction software, we could identify putative nuclear hormone receptor direct repeat candidate sites within the human and mouse *Muc2* promoter with the most conserved sites located at −2 kb (figure 6G,H).^35^ Therefore, we investigated NR2F6 binding capability to the mouse *Muc2* promoter via ChIP using scrapings of murine intestinal epithelial cells and specific PCR primers covering this *Muc2* promoter region. We found NR2F6 binding to the −2 kb region of the *Muc2* promoter in healthy colons (figure 6I); however, binding capability was found to be reduced under inflammatory DSS colitis conditions. Correlating with active chromatin histone H3ac (pan-acetyl) antibody binding was significantly enriched at this −2 kb region of the *Muc2* promoter when compared with histone H3K27me3 antibody which is mainly associated with transcriptional repression, but no differences between healthy and inflammatory state could be detected for either antibody (figure 6J,K).

As a next step and in order to prove human relevance, we analysed two human colon cancer cell lines well known to have different MUC2 secretion levels, namely, LoVo (high MUC2) and Caco-2 (low MUC2) cells. NR2F6 functionally binds to the distal promoter (−2 kb) region of the *MUC2* gene in LoVo but not in Caco-2 cells, as shown via ChIP using specific PCR primers covering the −2 kb region^32^ (figure 6L), suggesting that NR2F6:*MUC2* promoter interaction indeed results in altered transcriptional activity.

## Discussion

How NRs regulate gut homeostasis in the complex interplay between intestinal epithelial cells, the immune system and the microbiota is an area of active research. The aim of our current study was to clarify the role of the nuclear receptor NR2F6 in healthy and inflamed colon.

Having shown that *Nr2f6-*deficient T cells produce enhanced amounts of proinflammatory cytokines such as IFNγ, TNFα or IL-17^26–28^ after activation, we speculated on an immune cell intrinsic role of NR2F6 during mucosal injury within the GI tract. Other NRs such as the VDR are protective during colitis in CD4^+^ Th17 T cells; however, *VDR-*deficient CD8^+^ T cells, especially in combination with naive CD4^+^ T cells, result in aggravated colitis in *Rag-*deficient recipients due to enhanced proliferation and increased IFNγ and IL-17 levels in the gut.^36 37^ Surprisingly, our results showed that the loss of *Nr2f6* in CD4^+^ T and other immune cells enhanced inflammation neither in the transfer colitis model nor in BM chimeras.

Instead, our study identifies NR2F6 as a protective transcriptional regulator in the epithelial compartment regulating *Muc2* expression and subsequently intestinal permeability. In parallel to the major constituent of the mucus layer *Muc2*, the multidrug resistance 1 (*MDR1)* and *NEMO* which have been established to be important for epithelial barrier function, loss of NR2F6 led to spontaneous colitis development in aged mice.^38^

Our observations are in agreement with studies reporting that NRs play protective roles in intestinal epithelial integrity, as a significant decrease in the levels of mRNAs encoding, for example, VDR, HNF4α, MR, PPARγ and PXR has been demonstrated in intestinal samples from patients with IBD.^10 14^ In mice, deletion of the VDR increases mucosal injury that leads to high mortality in DSS-induced experimental colitis.^10^ In parallel, the activation of the FXR prevents chemically induced intestinal inflammation, improves colitis symptoms, inhibits epithelial permeability and reduces goblet cell loss.^15^ Intestinal steroidogenesis controls PPARγ expression in the colon, and this axis is impaired in UC.^13^

Both NR2F1 and NR2F2 have functionally important but different roles within the colon. NR2F1 (COUP-TFI), together with the inositol-requiring enzyme 1, suppresses microsomal triglyceride transfer protein expression at a transcriptional and post-transcriptional stage in undifferentiated intestinal cells and thereby restricts apoB lipoprotein biosynthesis.^39^ COUP-TFII (NR2F2) directly regulates the transcription and expression of SNAIL1 in human colon cancer tissue and thereby correlates with the inhibition of the expression of adhesion molecules such as ZO-1, E-cadherin and β-catenin and subsequently metastatic potential of colorectal adenocarcinoma cells.^40^

Although COUP-TF family members can bind to the same hormone response elements, we did not detect alterations in the expression of adherence molecules in colonic sections in the absence of NR2F6. Mucus components, such as Muc2, are indispensable to intestinal homeostasis, and alterations of mucus thickness primarily reflect Muc2 secretion.^41^ Other NRs also regulate and primarily enhance mucin gene expression; however, a detailed analysis of their functional role within the *Muc* gene cluster is still elusive.^42^ A PPAR binding site in the proximal *Muc1* promoter acts as a basal silencer in the absence of PPARγ, and its cooperation with a composite upstream enhancer element is both necessary and sufficient for PPARγ-dependent induction of *Muc1*.^9^ Consistently, during steady state, loss of NR2F6 resulted in reduced *Muc1* expression in colonic epithelial cells. As reduced *Muc1* expression protects mice from DSS-induced colitis,^43^ this observation cannot be causative for the enhanced colitis sensitivity in the *Nr2f6*-deficient setting. The NR HNF4α regulates goblet cell maturation and binds to the upstream promoter region of the *Muc3* gene in vivo.^14 16^ In Hnf4α^ΔIEpC^ mice, the expression of *Muc3* is markedly decreased, while *Muc1* expression is increased, and *Muc4*, *Muc5ac*, *Muc5b* and *Muc6* are slightly increased indicating that several *Muc* genes harbour binding sites for NRs.^14^ In contrast to HNF4α gene ablation, however, loss of NR2F6 led to reduced *Muc1* expression and reduced *Muc2* levels but resulted in no difference in expression of *Muc3*, *Muc4* and *Muc5ac* in steady state. During DSS colitis, *Muc2* and *Muc3* expression was selectively reduced in *Nr2f6*-deficient colonic scrapings whereas *Muc1*, *Muc4* and *Muc5ac* expression was unaltered. Additionally, the partial reduction of goblet cells may additively account for the observed reduction of the mucus layer in *Nr2f6*-deficient mice.

Taken together, we provide strong experimental evidence that loss of NR2F6 results in an altered colonic mucus constitution and, subsequently, increased susceptibility to intestinal inflammation. In conclusion, our work has identified an unexpected role of NR2F6 in intestinal homeostasis.
