# Supplementary material for: Nuclear orphan receptor NR2F6 as a safeguard against experimental murine colitis
Source: Gut. 2017 Aug 4;67(8):1434–44. doi: 10.1136/gutjnl-2016-313466 (PMC6204953; doi:10.1136/gutjnl-2016-313466)
Supplement: Supplementary material 1 [file gutjnl-2016-313466supp001.pdf]

Supplementary Files

Material and Methods:

DSS colitis analysis

Body weight, the presence of occult or gross blood per rectum, and stool consistency were determined every day. Experiments were terminated on day 7, colons were removed and flushed with ice-cold phosphate-buffered saline (PBS). Samples were harvested, either fixed in 4% formalin for <24 hours and embedded in paraffin to provide sections for histological scoring, or pieces of colonic tissue were used for *ex vivo* analysis such as RNA isolation or FACS analysis.

Bone marrow chimeras

Six to eight−week−old *Nr2f6*$^{+/+}$ and *Nr2f6*$^{-/-}$ mice were irradiated with 10 Gray using a linear accelerator with an iridium-192 radioactive source. After 2−3 h, mice were reconstituted intravenously with 1 × 10^6 bone marrow cells derived from either *Nr2f6*$^{+/+}$ or *Nr2f6*$^{-/-}$ mice. Post irradiation, mice received 10 mg/L neomycin sulfate prophylactically *via* the drinking water, *ad libitum*, for 2 weeks.

Transfer colitis model

Colitis was induced *via* the transfer of either *Nr2f6*$^{+/+}$ or *Nr2f6*$^{-/-}$ naïve CD4$^+$ T cells into *Rag1*$^{/-}$ mice. CD4$^+$ T cells were isolated from spleens and lymph nodes of wild-type or *Nr2f6*$^{-/-}$ mice using the mouse CD4$^+$ T Cell Isolation Kit II (Miltenyi Biotec O. No. 130-090-860) and a subsequent sorting purification step on a FacsAria (BD-Biosciences) leading to a purity of >99% of CD4$^+$CD45RB^{high} -naïve T cells. 5 x 10^5 naïve CD4$^+$ T cells were injected intraperitoneally (i.p.) into *Rag1*$^{-/-}$ recipient mice, which were monitored regularly for signs of disease, weight loss, hunched posture and diarrhoea. Colitis scores were assessed by researchers blinded to sample identity according to a score described in the Supplementary Materials & Methods.

Histological procedures and scoring

Colons, including cecum and rectum, were removed, flushed with ice-cold PBS and

immediately fixed in 4% buffered formalin or in Carnoy's fixative (60% methanol, 30% chloroform, and 10% acetic acid). Fixed tissues were further transferred into an automatic tissue processor (Leica, Wetzlar, Germany). Sections of 5 µm were stained with hematoxylin and eosin (H&E) or periodic acid–Schiff (PAS). All slides were scanned on a Pannoramic Scan digital slide scanner and investigated using Pannoramic Viewer software (both 3DHistech, Budapest, Hungary) in a blinded fashion. Sections from DSS-treated mice were assessed as described previously[46]; Briefly, distal colon was scored on a scale of 0–4 based on % of colon involvement by inflammation, % of crypt loss, presence of lymphoid follicles, edema, erosions, and density of inflammatory cells. The scores display the sum of the parameters for a total severity score. Numbers of goblet cells were assessed on PAS-stained slides and expressed as percentage per intestinal epithelial cells along the crypt axis. Numbers of goblet cells were assessed on PAS-stained slides and expressed as percentage per intestinal epithelial cells along the crypt axis.

Swiss roll histology

Whole colons were flushed with ice-cold PBS, opened longitudinally, curled on a lead and immediately fixed in 4% buffered formalin or in Carnoy's fixative. After sectioning, all slides were stained with hematoxylin-eosin, scanned on a Pannoramic Scan digital slide scanner and investigated using Pannoramic Viewer software (both 3DHistech, Budapest, Hungary) in a blinded fashion by two independent investigators. Inflammatory activity was assessed using a semi-quantitative score as previously described with minor modifications[45]. Each of four histological criteria (polymorphonuclear infiltrate, mononuclear infiltrate, epithelial injury and epithelial hyperplasia) was determined as absent (0), mild (1), moderate (2), or severe (3). Each parameter was assigned an extent factor reflecting its overall involvement ranging from 1 (< 10%), 2 (10–25%), 3 (25−50%), and 4 (>50%).

16S rRNA fluorescence *in situ* hybridization (FISH) analysis

Feces−containing colonic tissue samples were fixed in Carnoy's solution. Tissue sections were deparaffinized and hybridized with a pan-bacterial Eub338 5'−FITC−GCT GCC TCC CGT AGG AGT probe. After washing, slides were counterstained and coverslipped using ProLong® Gold Antifade Reagent including 4',6−diamidino−2−phenylindole (DAPI, Life Technologies). Samples were viewed and imaged on a Zeiss AxioObserver.Z1 in combination with a LSM700

confocal laser scanning system and scored as described previously[33]. Only slides with clear and crisp hybridization signals were included into enumeration. Mean values and standard deviations were calculated for bacterial concentrations and measurements of mucus layer thickness.

Immunohistochemistry

4 µm sections were deparaffinized, rehydrated and antigens were retrieved by pressure cooking in antigen unmasking solution (Vector Laboratories, Burlingame, CA). Non−specific protein binding sites were blocked using 1% BSA. Slides were incubated with their respective primary and secondary (anti-rabbit, anti-rat IgG Alexa Fluor 488® or Alexa Fluor594) antibodies at RT for 1 hour (for immunofluorescence) or 4°C overnight (for immunohistochemistry). Slides were mounted with Dako fluorescent mounting medium (S3023), which includes DAPI staining. IHC analysis of colons was performed in triplicates of 4 biopsy specimens of *Nr2f6*$^{+/+}$ and *Nr2f6*$^{-/-}$ mice. Digital IHC images were acquired with an Axiovert 40 CFL (ZEISS) microscope.

The following antibodies were used for immunofluorescence and immunohistochemistry: Mucin 2 (H-300) rabbit pAb (Santa Cruz Biotechnology, Dallas, Texas), polyclonal rabbit anti-human CD3 (Dako, A 0452), monoclonal NK1.1 (Novus Biologicals, PK136, NB100-77528), monoclonal anti-ZO-1 (Merck Millipore, R40.73, MABT11), E-Cadherin (24E10) rabbit mAB (Cell Signaling, #3195).

***In vivo* BrdU labeling assay**

For proliferation assays, animals were injected with 2.5 mg bromodeoxyuridine (BrdU, Becton Dickinson, Franklin Lakes, NJ) in 500 µl PBS. Colons were harvested after 2 or 24 hours. BrdU−positive nuclei were identified using a BrdU detection kit (Becton Dickinson). Data are expressed as BrdU-positive nuclei per crypt axes normalized to total nuclei per crypt.

Apoptosis analysis by TdT−dTptosis dUTP nick end labeling (TUNEL)

TUNEL staining was performed using the ApopTag kit (Roche, Basel, Switzerland) according to the manufacturer’s instructions. TUNEL-positive cells were counted in 5 randomly selected crypts per animal and expressed as the ratio to total intestinal epithelial cells (IEC).

Six mice from each group were studied.

Isolation of lamina propria lymphocytes (LPLs)

LPLs were obtained from colons of 8 to 12-week-old wild-type and *Nr2f6*$^{-/-}$ mice. Colons were removed, flushed with ice-cold PBS, opened longitudinally, cut into 5 mm pieces, and shaken for 20 min in Ca^{2+}/Mg^{2+} −free HBSS containing 10% FCS, 2 mM EDTA and 1 mM DTT. Next, fragments were vortexed at maximum speed and the supernatants (containing IELs) were transferred into fresh tubes. The remaining fragments were digested in RPMI 20% FCS, 100U/ml type IV Collagenase and fresh 20U/ml DNAse II and rotated at 37°C for 60 min. Thereafter, the remaining tissue fragments were re-suspended and sequentially passed through 100, 70, to 40 µm cell strainers. LPLs were pelleted by centrifugation, counted and stained for FACS analysis.

Flow cytometry

LPLs, lymph nodes and spleens were incubated with FcR Block (BD Biosciences, 553142) to prevent nonspecific antibody binding before staining with appropriate surface antibodies for 30 min at 4°C and washed with PBS+2% FCS. After the final wash step, cells were acquired on a BD LSRFortessa equipped with a violet, blue, and red laser. Appropriate single−stained cells were included to set up compensation protocols. Data were analysed using the FlowJo software (Tree Star Inc, OR, USA). The gating strategy is outlined in Supplementary figure2C. The following antibodies were used for flow cytometry: CD4-V500 (BD, 560783), CD4-PE (BD, 553049), CD8a-APC (BD, 553035), CD11c-PE-Cy7 (BD, 558079), CD45-V500 (BD, 561487), CD11b-PE (BD, 557397), CD45-FITC (eBiosciences, 11-0451-82), CD8a-PerCP Cy5.5 (eBiosciences, 45-0081-82), DX-5-PE-Cy7 (eBiosciences, 25-5971-81), TCR γδ-APC (eBiosciences, 17-5711-82), Ly-6C-APC (eBiosciences, 17-5932-82), CD45-APC (eBiosciences, 17-0451-81), CD3-PE-Cy7 (eBiosciences, 25-0031,82), FoxP3-FITC (eBiosciences, 11-5773-82), CD3-PE (eBiosciences, 12-0031-83), CD8a-bv421 (BioLegend, 100738), F4/80-PE-Cy7 (BioLegend, 123113), Gr-1-FITC (BioLegend, 108405), CD11b-APC (BioLegend, 101211).

Supplementary Figures

Figure 1 Supplementary

Klepsch V. et al.

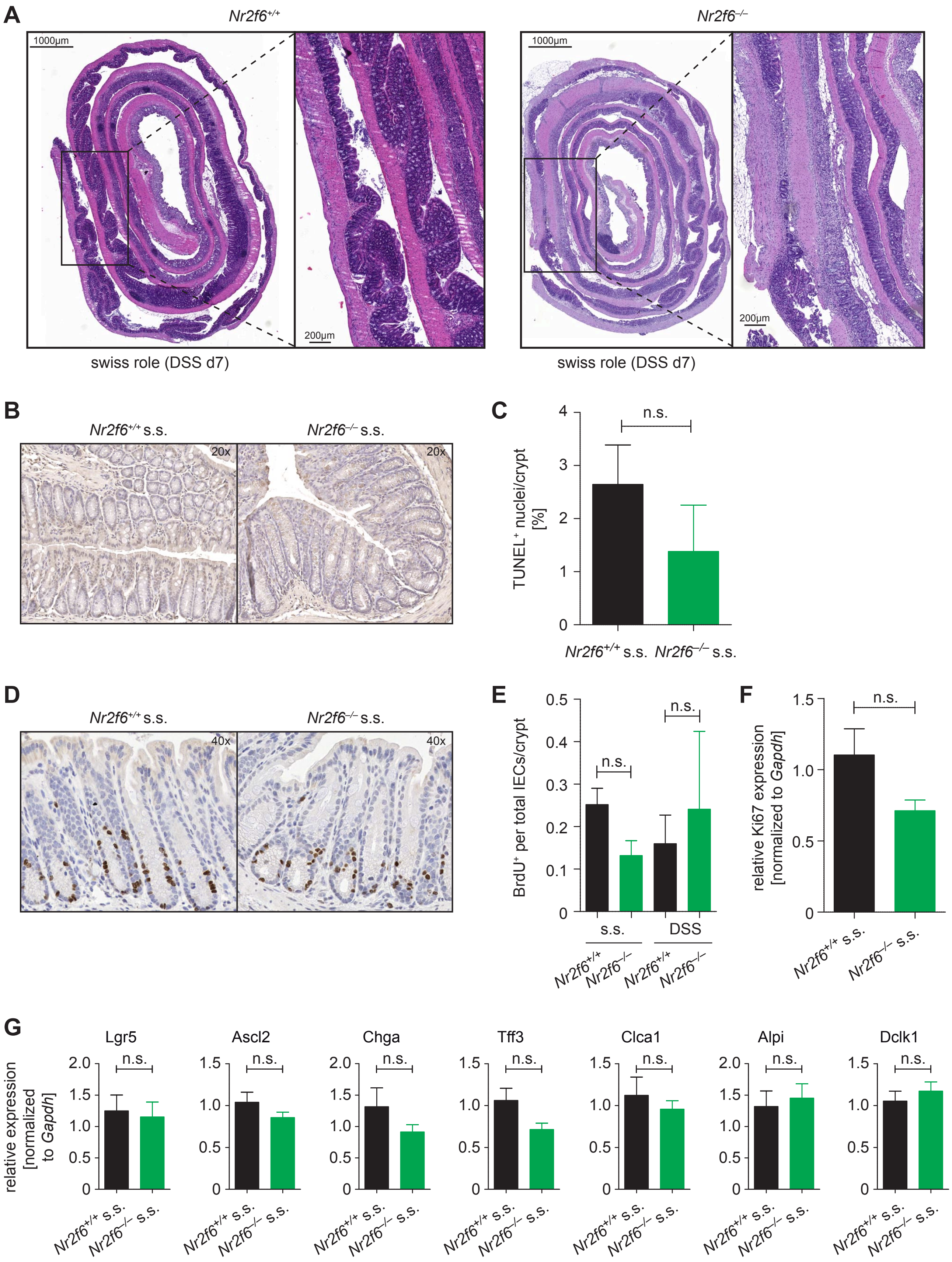

S-Figure 1 (A) *Nr2f6*$^{-/-}$ mice are more susceptible to DSS-induced colitis. Representative H&E stained sections of colonic Swiss rolls of *Nr2f6*$^{+/+}$ and *Nr2f6*$^{-/-}$ mice at day 7 of the experimental period of acute DSS-induced colitis. (B) Apoptosis in representative pictures of TUNEL-stained histology samples from steady state *Nr2f6*$^{+/+}$ and *Nr2f6*$^{-/-}$ colons (n=6). (C) Descriptive statistical analysis revealed no significant difference in TUNEL-positive nuclei per crypt. (D) Colon cell proliferation of steady state or inflamed (DSS d3) *Nr2f6*$^{+/+}$ and *Nr2f6*$^{-/-}$ mice determined by BrdU staining (staining cell nuclei in brown). (E) Quantification of BrdU-positive cells per crypt in steady state and DSS diseased wild-type and *Nr2f6*-deficient colons revealed no differences between genotypes. (F) Relative gene expression of the proliferation marker *Ki67* was unaltered between genotypes in steady state. (G) Steady state differentiation potential of intestinal epithelial cells in *Nr2f6*$^{+/+}$ and *Nr2f6*$^{-/-}$ colons was analyzed via qRT-PCR evaluating *Lgr5* and *Ascl2* for stem cells, *Chga* for enteroendocrine cells, *Tff3* and *Clca1* for goblet cells, *Alpi* (p=0.70) for enterocytes, and *Cdlk1* for tuft cells. Data are presented as mean $\pm$ SEM error bars and are representative of at least two independent experiments. Unpaired Student´s t test * $p<0.05$.

Figure 2 Supplementary

Klepsch V. et al.

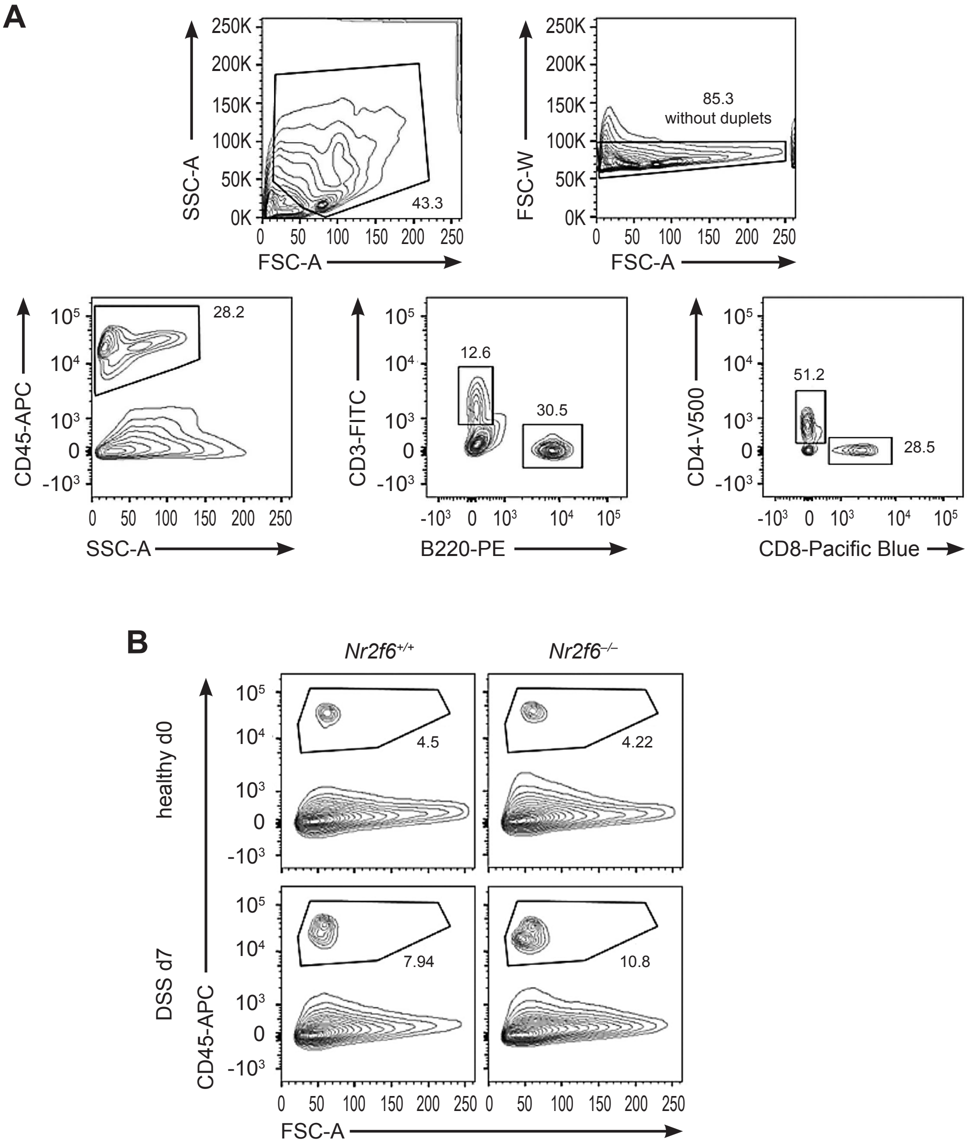

S-Figure 2 (A) Gating strategy for flow cytometry of colonic immune subsets after exclusion of doublets. (B) Basic characterization of $CD45^+$ leukocytes by flow cytometry in steady state *Nr2f6*$^{+/+}$ and *Nr2f6*$^{-/-}$ mice as well as on day 7 after DSS treatment. Numbers in quadrants indicate percent of parent population.

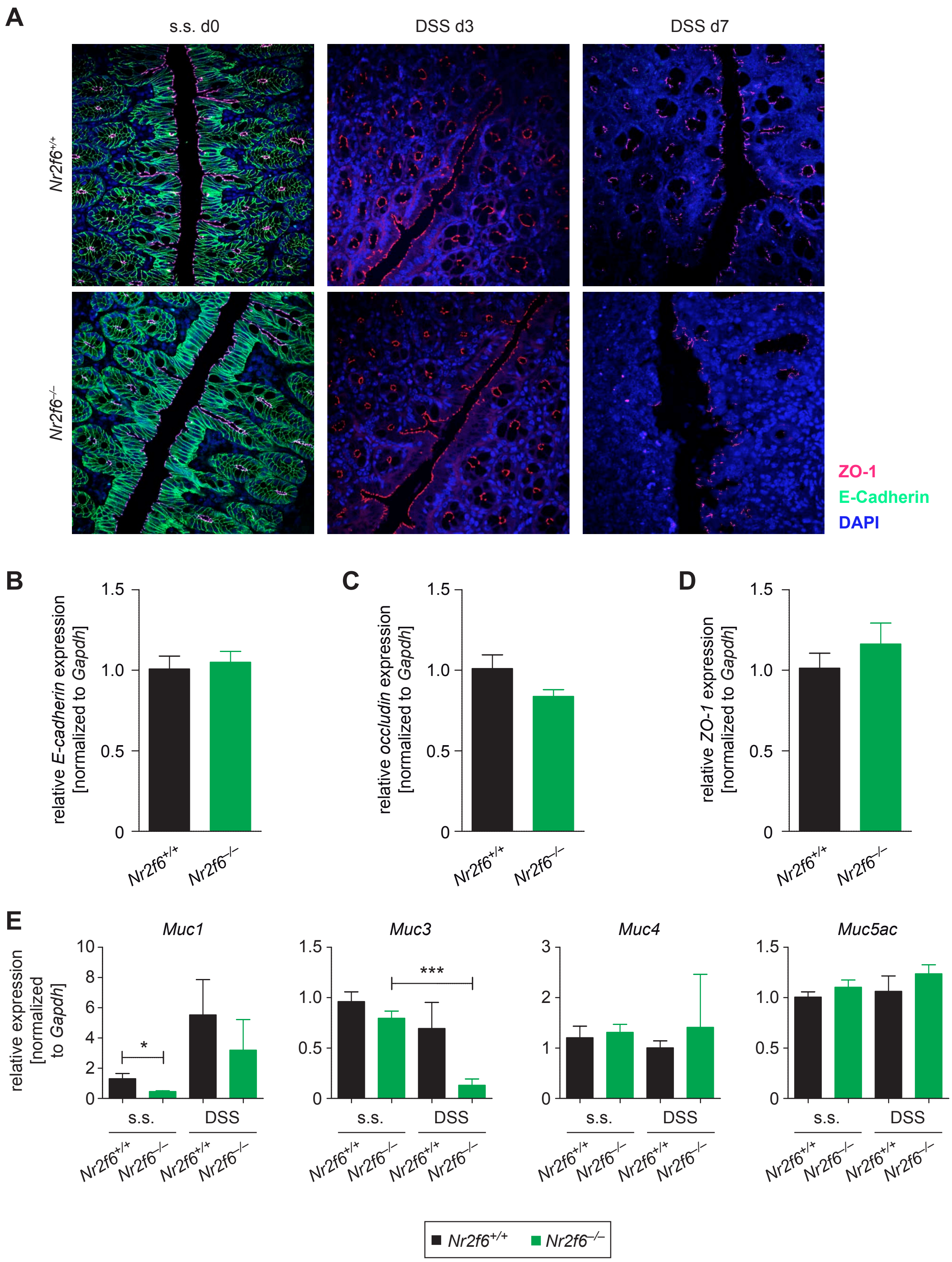
A
s.s. d0
DSS d3
DSS d7
Nr2f6+/+
Nr2f6–/–
ZO-1
E-Cadherin
DAPI
B
relative E-cadherin expression [normalized to Gapdh]
C
relative occludin expression [normalized to Gapdh]
D
relative ZO-1 expression [normalized to Gapdh]
1.5
1.0
0.5
0
Nr2f6+/+
Nr2f6–/–
E
relative expression [normalized to Gapdh]
Muc1
Muc3
Muc4
Muc5ac
*
***
s.s.
DSS
Nr2f6+/+
Nr2f6–/–

S-Figure 3 Analysis of tight junction, adherens junction and expression of *Muc* genes in *Nr2f6*-deficient colons. (A) Colon sections stained with DAPI (blue) and ZO-1 (red) as well as in the steady state with E-Cadherin (green). Analysis of mRNA expression of different junction markers *E-cadherin* (B), *occludin* (C) and ZO-1 (D) in colonic mucosal scrapings of *Nr2f6*$^{+/+}$ and *Nr2f6*$^{-/-}$ mice (n = 8). (E) *Muc* gene expression in *Nr2f6*$^{+/+}$ and *Nr2f6*$^{-/-}$ intestinal epithelial cell scrapings was analyzed via qRT-PCR in steady state and during DSS colitis (d7). Gene expression was investigated in steady state and during DSS colitis (d7). *Muc1* expression was different in steady state (p=0.019), but no altered expression of *Muc3* , *Muc4* and *Muc5ac* was detected in *Nr2f6*-deficient scrapings when compared with wild-type controls. Data are presented as mean ± SEM error bars and are representative of at least two independent experiments. Unpaired Student´s t test * p<0.05.
